# Performance of the Bebé VieScope Versus Direct Laryngoscopy During Pediatric Cardiopulmonary Resuscitation: A Prospective Randomized Simulation Study

**DOI:** 10.3390/children13010137

**Published:** 2026-01-17

**Authors:** Pawel Wieczorek, Halla Kaminska, Michal Pruc, Wojciech Wieczorek, Katarzyna Karczewska, Jacek Smereka, Şahin Çolak, Lukasz Szarpak

**Affiliations:** 1 Pediatric Intensive Care Unit (PICU), John Paul II Upper Silesian Health Centre in Katowice, 40-752 Katowice, Poland; 2 Department of Children’s Diabetology and Lifestyle Medicine, Faculty of Medical Sciences, Medical University of Silesia, 40-752 Katowice, Poland; 3 Institute of Medical Sciences, The John Paul II Catholic University of Lublin, 20-708 Lublin, Poland; 4 Department of Emergency Medicine, Medical University of Warsaw, 02-097 Warsaw, Poland; 5 Clinical Department of Anesthesiology, Intensive Care, and Pain Management, Mazovian Specialist Hospital, 26-617 Radom, Poland; k.karczewska@interia.eu; 6 Department of Emergency Medical Service, Wroclaw Medical University, 50-367 Wroclaw, Poland; 7 Department of Emergency Medicine, Haydarpaşa Numune Training and Research Hospital, 34668 Istanbul, Türkiye; dr.sahincolak@gmail.com; 8 Technology Transfer Center, The John Paul II Catholic University of Lublin, 20-708 Lublin, Poland; 9 Henry JN Taub Department of Emergency Medicine, Baylor College of Medicine, Houston, TX 77030, USA

**Keywords:** airway management, direct laryngoscopy, endotracheal intubation, intubation time, medical simulation, pediatric cardiopulmonary resuscitation, success rate, tubular optical laryngoscope, VieScope laryngoscope

## Abstract

**Highlights:**

**What are the main findings?**

- In a simulated pediatric cardiac arrest model, the VieScope laryngoscope had better first-pass success rates and shorter intubation times than the Macintosh and Miller direct laryngoscopes, especially when chest compressions were not stopped.
- Performance differences between devices were minor without chest compressions but became significant during dynamic resuscitation conditions, with VieScope consistently offering better glottic visualization and procedural efficiency.

**What are the implications of the main findings?**

- Optical tubular laryngoscopes may be helpful as airway management tools during pediatric cardiopulmonary resuscitation, particularly when reducing interruptions to chest compressions is important.
- Integrating such devices into pediatric airway training and resuscitation protocols could improve intubation performance in difficult, high-movement situations, pending validation in clinical studies.
- As these results were obtained in a controlled simulation setting, prospective clinical studies are needed to determine whether the observed performance differences translate into improved airway management and outcomes during real pediatric cardiopulmonary resuscitation.

**Abstract:**

**Background/Objectives**: Effective airway management during pediatric cardiopulmonary resuscitation (CPR) is crucial but technically challenging, especially during continuous chest compressions. While direct laryngoscopy with Macintosh (MAC) or Miller (MIL) blades remains the standard, optical devices such as the VieScope (VSL) may enhance performance under dynamic resuscitation conditions. This study compared first-pass success and intubation time, as well as procedural difficulty and glottic visualization, of MAC, MIL, and VSL during simulated pediatric cardiopulmonary resuscitation. **Methods**: This prospective, randomized crossover simulation study involved 53 medical students. Participants performed endotracheal intubation on a high-fidelity manikin simulating a 5-year-old pediatric patient using MAC, MIL, and the Bebé VieScope laryngoscope. Each technique was evaluated in two scenarios: with and without continuous chest compressions. **Results**: Without chest compressions, first-pass success (FPS) and intubation time varied significantly between techniques. VSL achieved the highest FPS (100%; *p* = 0.032) and the shortest intubation time (27.9 ± 9.2 s; *p* = 0.040), performing faster than MIL and achieving higher FPS than MAC. Visualization quality, ease of intubation, and optimization maneuvers were similar across techniques. During continuous chest compressions, all outcomes differed significantly. FPS increased from MAC to MIL and VSL (*p* = 0.001), with MAC showing the lowest success rate. VSL showed the shortest intubation time (35.9 ± 13.0 s; *p* < 0.001), better glottic visualization, easier intubation, and fewer optimization maneuvers, followed by MIL. **Conclusions**: In this simulated pediatric cardiac arrest model, the VieScope laryngoscope demonstrated superior overall performance, especially during uninterrupted chest compressions. Optical tubular laryngoscopy may therefore provide clinically relevant benefits in pediatric resuscitation where maintaining high-quality chest compressions is crucial. Given the manikin-based design of this study, confirmation of these findings in clinical pediatric cardiac arrest settings will require further prospective clinical investigation.

## 1. Introduction

Effective airway management in pediatric patients during cardiopulmonary resuscitation (CPR) is crucial for advanced resuscitation and significantly affects survival rates and neurological outcomes after cardiac arrest (CA) [1,2]. Endotracheal intubation remains the gold standard for maintaining airway patency, preventing aspiration, and facilitating accurate mechanical ventilation, especially when bag–valve–mask ventilation is ineffective or impossible.

In the pediatric population, intubation is especially challenging due to differences in anatomy, smaller airway diameter, susceptibility to rapid deterioration, and the need to minimize interruptions in chest compressions. The guidelines of the American Heart Association (AHA) and the American Academy of Pediatrics emphasize that the choice of advanced airway management technique should be tailored to the personnel’s experience, equipment availability, and the specifics of the clinical situation [3].

In recent years, there has been intensive development of new intubation tools, such as video laryngoscopes (e.g., McGrath, GlideScope, AirTraq, UEScope, Coopdech, King Vision) and innovative devices, including Bebé VieScope laryngoscope (VSL) [4]. Simulation studies indicate that video laryngoscopes and the VieScope provide superior glottic visualization compared with conventional Macintosh (MAC) and Miller (MIL) laryngoscopes [5,6,7]. This improved view translates into higher first-pass intubation success rates and shorter procedure times [8,9]. These advantages are most pronounced in difficult airway scenarios and when intubation is performed during uninterrupted chest compressions [10,11,12]. In particular, VieScope demonstrates an advantage over direct laryngoscopes in scenarios involving tongue swelling and during continuous resuscitation, offering better glottis visibility and higher first-attempt success rates [4,5,6]. On the other hand, in situations such as massive gastric aspiration, the classic MAC may provide faster airway protection and a lower risk of aspiration than the VieScope [13]. Research findings also indicate that flexible-tip laryngoscopes (e.g., TMAC) and selected video models can significantly improve intubation success rates in difficult airway conditions [12,14].

Despite technological advances, selecting the best intubation tool for pediatric resuscitation remains an active area of research. There is a need for additional comparative studies on the effectiveness, procedure time, safety, and ergonomics of VSL, MAC, and MIL in simulated CA conditions in children. Such research is essential to develop evidence-based guidelines that can enhance the quality of emergency care for the youngest patients.

This study aims to comprehensively compare direct laryngoscopy with a MAC, direct laryngoscopy with a MIL, and optical VSL in simulated pediatric CPR under both with- and without-chest-compression scenarios, taking into account the latest literature data and scientific society guidelines, to identify the most effective strategies for clinical practice.

## 2. Materials and Methods

### 2.1. Study Design

This study was designed as a prospective, randomized crossover trial conducted under high-fidelity medical simulation conditions. By adopting a crossover design, each participant applied all three endotracheal intubation techniques in the same controlled environment, minimizing the impact of individual differences between operators.

The study was conducted in accordance with the principles of the Declaration of Helsinki. The study protocol was approved by the Bioethics Committee of the John Paul II Catholic University of Lublin, Poland (approval no. 13/2025 KUL). The study was conducted in a high-fidelity medical simulation center at Jan Długosz University in Częstochowa, Poland. All participants were informed about the nature and objectives of the study and provided written informed consent before participation.

### 2.2. Participants

The study involved medical students at an advanced stage of their pre-diploma education who had completed Pediatric Advanced Life Support (PALS) training in accordance with American Heart Association guidelines. All training was delivered by AHA-certified instructors and included both theoretical instruction and hands-on simulation of pediatric cardiopulmonary resuscitation and basic airway management.

Participants had comparable levels of prior training and simulation exposure. Individuals were eligible for inclusion if they had completed the full PALS course and had no significant clinical experience with pediatric endotracheal intubation. Exclusion criteria included prior professional experience in anesthesiology, intensive care, or emergency medicine, participation in pediatric intubation during clinical practice, upper limb conditions that could impair intubation performance, or withdrawal of informed consent at any stage of the study.

The study excluded participants who had not completed PALS training according to AHA guidelines or who had not participated in the comprehensive initial training phase specified in the study protocol, as well as individuals with significant prior clinical experience in pediatric endotracheal intubation, including those in internships or employed in anesthesiology, intensive care, or emergency medicine departments. People who have or have had upper limb injuries (like fractures, immobilization, limited range of motion, or pain) that could stop or make it much harder to do endotracheal intubation correctly, as well as people who say they have other health problems that make it unsafe to do simulation procedures or who refuse to take part or withdraw their consent at any point in the study.

### 2.3. Intubation Techniques

**Figure 1:**
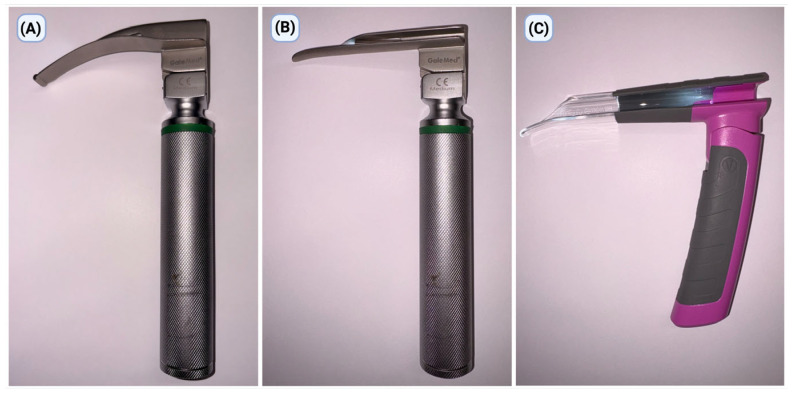
Laryngoscopes used in the study: (**A**) Macintosh laryngoscope (MAC), (**B**) Miller laryngoscope (MIL), and (**C**) bébé Vie Scope laryngoscope (VSL).

The study assessed three methods of endotracheal intubation with laryngoscopes used with blade sizes recommended by the manufacturers for a pediatric patient corresponding to a 5-year-old child, in alignment with contemporary pediatric airway care standards (Figure 1). All devices were chosen so that they could be tested for intubation effectiveness in a controlled simulation setting:

The Laryngoscope with Macintosh blade (MAC; GaleMed^®^, Yilan, Taiwan) was the standard method for performing direct laryngoscopy. The process used a size-2 MAC blade. This method used the tip of the blade to indirectly lift the epiglottis by putting it in the pyriform sinus. This made it possible to see the entrance to the larynx.

The Laaryngoscope with a Miller blade (MIL; GaleMed^®^, Yilan, Taiwan) was a straight-blade method of direct laryngoscopy that was often used in children. The study used a size 2 MIL spoon, facilitating direct elevation of the epiglottis and exposure of the laryngeal structures, which is especially critical in pediatric contexts given the epiglottis’s distinct position and morphology.

The VSL was employed as a different strategy to regulate the airway. It is a stiff, tubular optical laryngoscope made just for kids. It lets you see the laryngeal intake directly through a cylindrical lumen. This design makes it easier to line up the oral and pharyngeal channels. It enables you to see the laryngeal structures without having to do the usual curved or straight-blade laryngoscopic techniques. The item was used as recommended by the manufacturer for managing children’s airways.

A 5.0 mm uncuffed endotracheal tube was used for all intubation attempts without a stylet in order to standardize conditions across devices and to avoid the confounding effects of adjuncts that may preferentially facilitate certain laryngoscopic techniques.

### 2.4. Randomization and Study Protocol

Before the study began, all participants attended a standardized 60 min introductory training session to standardize their level of preparation and limit the impact of learning on the study results. The training included a review of the principles of advanced airway management in children, a discussion of techniques for using all laryngoscopes being evaluated, and an introduction to the simulation conditions in which the study was conducted.

The practical part of the training, lasting 30 min, was conducted using the Laerdal Resusci Junior QCPR pediatric simulator (Laerdal Medical, Stavanger, Norway), which allows for realistic simulation of CPR in children. During the training session, participants performed airway clearance attempts using MIL, MAC, and VSL, which allowed them to familiarize themselves with the characteristics of each technique before beginning the actual study.

One month after the training stage was completed, the study was conducted. Endotracheal intubation procedures were performed on a high-fidelity pediatric simulator (5-Year-Old Patient, PEDI^®^ Airway Trainer; Gaumard Scientific, Miami, FL, USA), set up on a flat surface under controlled lighting conditions. The simulator was set up to simulate CA in a 5-year-old child, reflecting a clinical situation that required advanced resuscitation in accordance with contemporary pediatric guidelines.

Each participant performed endotracheal intubation in two separate study scenarios:

- Scenario A—endotracheal intubation performed without chest compressions,
- Scenario B—endotracheal intubation performed during continuous chest compressions.

A LUCAS 3 mechanical chest compression device (Physio-Control, Lund, Sweden) was used in continuous compression mode to ensure consistent chest compressions across all situations. For each intubation attempt, chest compressions were delivered at a predefined rate and depth in accordance with the most recent pediatric resuscitation guidelines, ensuring consistent conditions across all attempts [15].

**Figure 2:**
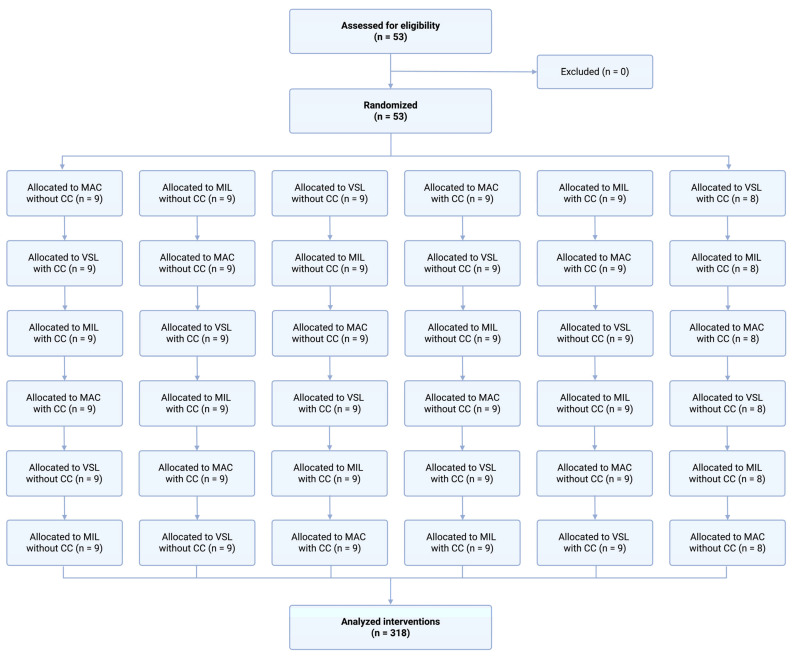
Randomization flow chart.

The order of laryngoscope use and the sequence of study scenarios were randomly assigned for each participant using a computerized random number generator. A cross-over design was used, in which each participant performed intubation using all three techniques in both scenarios (Figure 2).

Ten-minute rest breaks were provided between attempts to limit the impact of fatigue on the study results. Each participant had one intubation attempt for each laryngoscope scenario combination. Only one study participant was present in the simulation room during the procedures to reduce the risk of learning by observation.

### 2.5. Outcomes

The primary endpoint of the study was first-pass success (FPS). FPS was defined as the correct placement of the endotracheal tube in the trachea on the first attempt, without the need to withdraw the equipment or restart the procedure. The effectiveness of intubation was objectively confirmed by the simulator’s measurement system, which recorded correct lung ventilation.

Secondary endpoints included several parameters describing both the procedure’s effectiveness and technical difficulty. Intubation time was measured as the interval between insertion of the laryngoscope into the oral cavity and delivery of the first adequate ventilation, recorded in seconds. Visualization of the glottis was graded using the Cormack–Lehane classification [16]. To provide a more detailed assessment of laryngeal exposure, the percentage of glottic opening (POGO score) was also documented [17].

In addition to the objective measures, operators were asked to rate the ease of intubation using a visual analog scale, with higher scores indicating less difficulty. The number of optimization maneuvers required to successfully perform intubation, such as adjusting the head position, adjusting the laryngoscope, or performing additional manipulations to improve glottic visibility, was also recorded.

### 2.6. Statistical Analysis

An a priori sample size calculation was performed for the primary outcome, first-pass success, using an approach appropriate for a randomized crossover design with paired comparisons. The calculation was based on an expected clinically relevant difference in first-pass success between devices during uninterrupted chest compressions, with a two-sided significance level of 0.05 and a statistical power of 80%. The estimated minimum required sample size was 44 participants. To account for potential incomplete data, the target sample size was increased, and a total of 53 participants were ultimately included in the study.

Continuous variables are presented as means and standard deviations (SD), whereas categorical variables are reported as counts and percentages. Data normality was assessed using the Shapiro–Wilk test. Owing to the non-normal distribution of several variables and the ordinal nature of some outcomes, the Kruskal–Wallis test was used to compare more than two groups. When a statistically significant overall difference was identified, post hoc pairwise comparisons were conducted with appropriate correction for multiple testing to determine which group pairs accounted for the observed differences. All analyses were performed separately for each study scenario (intubation with and without chest compressions) and for each intubation technique evaluated. Statistical analyses were performed using Stata software, version 18 (StataCorp LLC, College Station, TX, USA). All statistical tests were two-sided, and a *p*-value < 0.05 was considered statistically significant.

## 3. Results

Fifty-three medical students (29 men, 54.7%) with no clinical or simulation experience in intubation using VSL participated in the study. All participants in the study claimed simulation experience with intubation using direct laryngoscopy.

### 3.1. Intubation Without Chest Compression Scenario

In the scenario without ongoing chest compressions, the number of successful FPS differed across groups, with values of 68.9%, 84.9%, and 100% for MAC, MIL, and VSL, respectively (Table 1).

**Figure 3:**
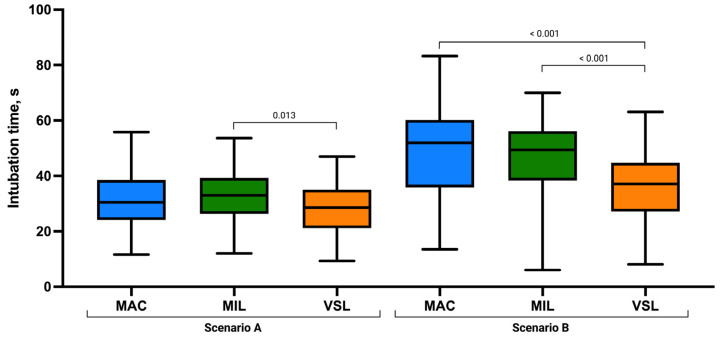
Endotracheal intubation time (seconds) using the Macintosh (MAC), Miller (MIL), and VieScope (VSL) laryngoscopes in two scenarios: without chest compressions (Scenario A) and during uninterrupted chest compressions (Scenario B). Data are presented as box-and-whisker plots showing the median, interquartile range, and minimum and maximum values.

The mean time to intubation was shortest in the VSL group (27.92 ± 9.18 s), compared with MIL (32.64 ± 9.31 s) and MAC (31.24 ± 9.95 s; Figure 3). Nonparametric analysis using the Kruskal–Wallis test demonstrated a statistically significant difference in intubation time among the three techniques (χ^2^ = 0.040, *p* = 0.040; Table 2).

**Figure 4:**
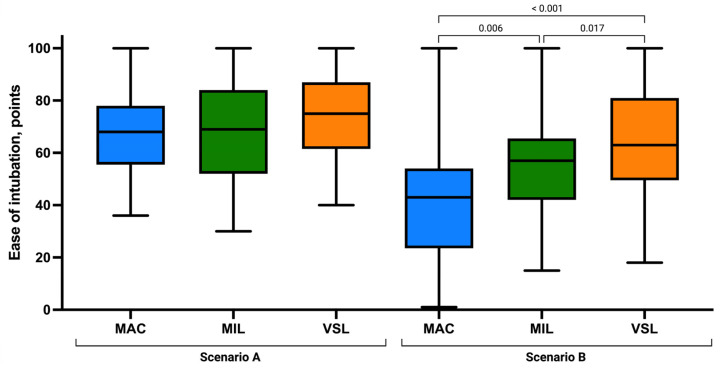
Ease of intubation using the Macintosh (MAC), Miller (MIL), and VieScope (VSL) laryngoscopes in two scenarios: without chest compressions (Scenario A) and during uninterrupted chest compressions (Scenario B). Data are presented as box-and-whisker plots showing the median, interquartile range, and minimum and maximum values.

Assessment of laryngeal view revealed comparable distributions of Cormack–Lehane grades across groups. Grade 1 visualization was most frequently observed in the VSL group (n = 31), followed by MIL (n = 26) and MAC (n = 22). No grade 4 views were recorded in any group. The mean POGO score was highest in the MIL group (87.38 ± 12.45), followed by VSL (86.17 ± 11.36) and MAC (83.36 ± 14.33), although these differences were not statistically significant (*p* = 0.346; Figure 4).

**Figure 5:**
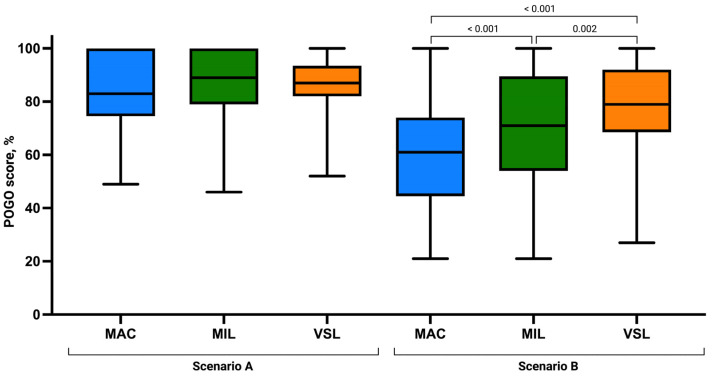
Percentage of Glottic Opening Score using the Macintosh (MAC), Miller (MIL), and VieScope (VSL) laryngoscopes in two scenarios: without chest compressions (Scenario A) and during uninterrupted chest compressions (Scenario B). Data are presented as box-and-whisker plots showing the median, interquartile range, and minimum and maximum values.

Ease of intubation scores were similar across techniques, with slightly higher mean values in the VSL group (73.53 ± 15.67) than in MIL (68.68 ± 19.04) and MAC (67.92 ± 15.96; Figure 5). The number of optimization maneuvers required was lowest in the VSL group (0.68 ± 0.83), followed by MIL (0.92 ± 0.92) and MAC (1.15 ± 1.09). However, no statistically significant differences were identified for ease of intubation (*p* = 0.189) or the need for optimization maneuvers (*p* = 0.202).

Overall group comparisons demonstrated that statistically significant differences were confined to intubation time (*p* = 0.040) and FPS (*p* = 0.032), whereas all visualization- and difficulty-related parameters remained comparable across techniques (Table 2). Post hoc pairwise comparisons demonstrated that the significant difference in intubation time was attributable to a shorter time in the VSL group compared with the MIL group (*p* = 0.013), while comparisons between MAC and MIL (*p* = 0.366) and between MAC and VSL (*p* = 0.111) did not reach statistical significance. For FPS rate, post hoc analysis revealed a statistically significant difference between the MAC and VSL groups (*p* = 0.017). Differences between MAC and MIL (*p* = 0.063) and between MIL and VSL (*p* = 0.566) were not statistically significant. No statistically significant pairwise differences were observed in post hoc analyses for POGO score, Cormack–Lehane grade, ease of intubation, or optimization maneuvers (all *p* > 0.05).

### 3.2. Intubation with Uninterrupted Chest Compression Scenario

In the scenario involving ongoing chest compressions, marked differences were observed between the analyzed intubation techniques. The number of successful first-pass intubations increased progressively from MAC (52.8%) to MIL (73.6%) and VSL (84.9%). The mean time to successful intubation was longest with MAC (49.24 ± 16.05 s), intermediate with MIL (46.86 ± 14.08 s), and shortest with VSL (35.85 ± 12.95 s; Table 3).

The Kruskal–Wallis analysis demonstrated statistically significant differences between groups for all assessed parameters, including time to intubation (*p* < 0.001), FPS *p*-value (*p* < 0.001), POGO score (*p* < 0.001), Cormack–Lehane grade (*p* < 0.001), ease of intubation (*p* < 0.001), and the number of optimization maneuvers required (*p* = 0.035).

Post hoc analysis revealed that the reduced intubation time associated with the VSL technique was the primary driver of the overall significance, with VSL performing significantly faster than both MAC and MIL (both *p* < 0.001). No statistically significant difference in intubation time was identified between MAC and MIL (*p* = 0.509). For first-pass success, MAC was associated with significantly lower success compared with both MIL (*p* = 0.027) and VSL (*p* < 0.001), whereas no significant difference was observed between MIL and VSL (*p* = 0.151). Beyond first-pass success, the VieScope demonstrated superior overall performance, including shorter intubation time, improved glottic visualization, greater ease of intubation, and fewer optimization maneuvers compared with both direct laryngoscopy techniques.

Visualization of the glottis differed substantially between techniques. POGO scores and Cormack–Lehane grades consistently favored VSL, followed by MIL, with MAC providing the least favorable laryngeal view. Post hoc analysis confirmed a consistent advantage of VSL over MAC and MIL in glottic visualization parameters.

Subjective ease of intubation also varied significantly across the three methods, with VSL rated the easiest, followed by MIL and MAC. All pairwise comparisons were statistically significant (MAC vs. MIL: *p* = 0.006; MAC vs. VSL: *p* < 0.001; MIL vs. VSL: *p* = 0.017), indicating meaningful differences in procedural difficulty under continuous chest compressions.

The requirement for optimization maneuvers was highest in the MAC group and lowest in the VSL group. Post hoc testing demonstrated a statistically significant difference only between MAC and VSL (*p* = 0.019), while comparisons between MAC and MIL (*p* = 0.392) and between MIL and VSL (*p* = 0.065) did not reach statistical significance (Table 4).

## 4. Discussion

In modern pediatric CPR guidelines, airway management must be balanced with the need to maintain high-quality chest compressions with minimal interruptions. They often suggest that breaks should last no more than 10 s, which makes tracheal intubation during compressions very difficult [3,18,19]. In this prospective, randomized crossover simulation study, PALS-trained medical students used three devices to perform endotracheal intubation on a standardized 5-year-old pediatric airway model: direct laryngoscopy with a MAC, direct laryngoscopy with a MIL, and the optical VSL. They did this under two conditions: without chest compressions (Scenario A) and with chest compressions continuously (Scenario B).

### 4.1. Principal Findings

The principal finding of this study is that device-related performance differences were modest in a static airway (Scenario A) but became pronounced during uninterrupted chest compressions (Scenario B).

In Scenario A (no compressions), VSL achieved the highest FPS (FPS: 53 vs. 45 for MIL and 37 for MAC) and the shortest mean intubation time (27.92 ± 9.18 s vs. 32.64 ± 9.31 s for MIL and 31.24 ± 9.95 s for MAC). Overall, group differences were statistically significant for FPS (*p* = 0.032) and intubation time (*p* = 0.040). In contrast, visualization metrics (Cormack-Lehane grade, POGO) and difficulty-related measures (ease of intubation, optimization maneuvers) were comparable across techniques.

Although participants reported prior simulation exposure to Macintosh laryngoscopy, this exposure likely reflects limited training rather than competency in pediatric direct laryngoscopy. In a pediatric airway, the Macintosh technique relies on indirect epiglottic elevation and precise alignment; these steps may be less forgiving for novice operators, even in a static setting. In contrast, the Miller blade and the tubular optical device may provide more direct epiglottic control and a more reproducible laryngeal view, which may partially explain the lower first-pass success observed with Macintosh in our cohort.

In Scenario B (continuous compressions), all assessed outcomes differed significantly between techniques. VSL produced the shortest time to intubation (35.85 ± 12.95 s vs. 46.86 ± 14.08 s for MIL and 49.24 ± 16.05 s for MAC) and the highest FPS (45 vs. 39 for MIL and 28 for MAC). VSL was also associated with better glottic visualization (higher POGO scores and more favorable Cormack–Lehane grades), higher subjective ease-of-intubation ratings, and fewer optimization maneuvers compared with MAC; MIL generally performed intermediate between VSL and MAC, with obvious advantages over MAC in FPS and visualization.

From a clinical process perspective, the magnitude of the time difference under compressions (≈11–13 s faster with VSL than with MIL/MAC) is potentially meaningful because prolonged airway attempts during CPR can increase cognitive load, degrade coordination, and raise the likelihood of unplanned pauses or deterioration in compression quality even if interruptions are not formally intended [20,21,22].

### 4.2. Relationship to Prior Research

These findings are consistent with simulation literature suggesting that devices designed to maintain a stable laryngeal view under dynamic conditions, such as videolaryngoscopes/video stylets and tubular optical laryngoscopes (e.g., the VSL), may achieve higher FPS and improved glottic visualization than conventional direct laryngoscopy during ongoing CPR [4,6,21,23,24,25]. Meta-analyses and randomized trials have shown that videolaryngoscopy is associated with improved glottic visualization and, in selected resuscitation settings, higher FPS compared with direct laryngoscopy, particularly under conditions such as uninterrupted chest compressions [21,23,24]. Consistent with these observations, pediatric simulation studies have reported superior performance of the VieScope compared with MAC during chest compressions, including improved visualization and subjective ease of intubation [4,6].

A plausible explanation for the observed performance advantage relates to several complementary mechanisms. First, both videolaryngoscopes and tubular optical laryngoscopes tend to provide a more stable and reproducible view of the larynx, one that is less easily disrupted by the head and chest movement generated during ongoing chest compressions. In addition, these devices appear to be less dependent on precise anatomical alignment, allowing effective visualization even when optimal head and neck positioning cannot be achieved during CPR. Finally, particularly for less experienced operators, the use of such devices may reduce cognitive and physical workload, leading to fewer optimization maneuvers and more efficient task completion in high-stress resuscitation settings [21,23,24,25].

Available simulation evidence suggests that these device characteristics may confer procedural advantages during pediatric CPR under dynamically unfavorable conditions; however, translation of these findings into clinical outcome benefits requires further prospective investigation [4,6,10,21,22,23,24,25,26].

### 4.3. Interpretation in the Context of Pediatric Intubation Literature

The interpretation of these results should be placed within the context of current evidence on pediatric airway management. In controlled pediatric settings, involving mainly patients with normal airway anatomy, large meta-analyses and systematic reviews have shown that improved visualization of the glottis with video laryngoscopy does not always translate into a higher rate of successful intubation on the first attempt; the benefits depend on the clinical context [25,27,28,29,30]. The results of scenario A are consistent with these observations: visualization parameters were similar across devices, and significant differences were observed only in first-attempt success rates and intubation times, with the most pronounced clinical differences occurring under conditions dynamically complicated by continuous chest compressions [4,31,32].

From a practical standpoint, no single device is optimal for every situation. When the airway is markedly contaminated, as in scenarios simulating massive gastric regurgitation, the MAC has allowed faster intubation than the VieScope. In these circumstances, the ability to suction quickly and maintain a clear field appears to favor traditional laryngoscopic approaches [13]. In contrast, for difficult airways, the PeDI registry and the American Society of Anesthesiologists guidelines indicate a clear advantage of video laryngoscopy in terms of first-attempt success and reduced complications [31,33].

In pediatric resuscitation, the AHA and American Academy of Pediatrics guidelines emphasize that advanced airway management techniques can improve ventilation and reduce the risk of aspiration. Still, their implementation requires a high level of competence and may interrupt chest compressions, which is undesirable [3]. It is also worth noting that in the neonatal and infant populations, video laryngoscopy may increase the likelihood of a successful first attempt and reduce the risk of complications. However, it does not shorten the time to intubation [28,30].

The results of this study support the concept that the advantage of videolaryngoscopy over direct techniques becomes most apparent under dynamically challenging conditions, such as uninterrupted chest compressions or difficult airway scenarios. In contrast, under routine pediatric conditions, the benefits appear more limited and remain highly context dependent. The choice of technique should be individualized, taking into account the potential benefits and limitations of each method and the team’s competence [3,4,13,25,27,28,29,30,31,33].

### 4.4. Direct Laryngoscopes During Continuous Compressions

During continuous chest compressions, the MIL generally outperformed the MAC in terms of first-attempt intubation success and laryngeal visualization quality. In the present study, MAC was associated with significantly lower first-attempt success rates than MIL. In contrast, MIL did not differ considerably from VSL, although VSL achieved shorter intubation times and more favorable visualization parameters.

The design of the MIL allows the operator to lift the epiglottis directly, an advantage that becomes especially apparent during pediatric resuscitation, where time pressure and chest movement leave little room for precise optimization. Greater control over the epiglottis may translate into better exposure of the glottis and, in turn, a higher likelihood of successful intubation on the first attempt. This observation is supported mainly by simulation data and reflects known characteristics of pediatric airway anatomy [34,35,36]. When chest compressions are maintained without interruption, the advantage of the MIL over the MAC tends to become more apparent, particularly in terms of visualization and FPS, which may help avoid unintended pauses during CPR [9,37,38].

Taken together, the results indicate that the MIL provides practical advantages over the MAC for pediatric intubation during resuscitation. At the same time, in this study, VSE showed the most favorable overall performance, particularly with respect to intubation speed, quality of visualization, and perceived ease of the procedure [38].

### 4.5. Clinical and Educational Implications

In pediatric CPR, airway management rarely occurs under ideal conditions. When chest compressions continue without interruption, even minor losses in visualization can quickly translate into failed attempts. Simulation data suggest that devices capable of maintaining a stable laryngeal view under these circumstances may improve FPS and reduce procedural friction [4,8,30,39,40].

Tubular optical laryngoscopes, including the VSL, belong to a distinct group of airway devices that provide direct optical visualization through a rigid tubular lumen, rather than relying on indirect video imaging. Simulation studies point to a practical advantage of these devices during resuscitation with continuous chest compressions. Ongoing movement tends to disrupt visualization, yet a more stable line of sight may help offset this effect. In pediatric models, tubular optical laryngoscopes have been associated with improved glottic exposure and a lower need for repeated optimization compared with curved-blade direct laryngoscopy [2,3,4].

The optical tubular design of the VieScope differs fundamentally from both conventional direct laryngoscopes and videolaryngoscopes. Rather than relying on indirect visualization through blade-mediated displacement of soft tissues or on a video camera positioned near the blade tip, the VieScope provides a rigid, cylindrical optical channel that establishes a direct line of sight to the laryngeal inlet. This geometry promotes a midline approach and aligns the operator’s visual axis with the tracheal axis early during insertion.

During ongoing chest compressions, repetitive vertical movement of the thorax and head can disrupt blade position and degrade glottic exposure when using conventional direct laryngoscopy. In contrast, the rigid tubular structure of the VieScope may dampen the effect of this motion by stabilizing the visual axis within the oropharynx and reducing dependence on precise head–neck alignment. Once positioned, the tubular lumen maintains a consistent view of the laryngeal structures, which may explain the improved glottic visualization and reduced need for optimization maneuvers observed in this study.

These design characteristics may be particularly advantageous for less experienced operators, for whom maintaining optimal laryngoscopic alignment during dynamic resuscitation conditions can be challenging. By providing a more reproducible visual pathway under motion, the optical tubular design may help preserve visualization quality during uninterrupted chest compressions.

From an educational perspective, tubular optical laryngoscopes may serve as a valuable adjunct in pediatric airway management training. Their optical design encourages deliberate midline insertion, direct epiglottic control, and explicit understanding of airway anatomy, which are fundamental skills in pediatric intubation. Simulation-based studies involving novice providers indicate that training with tubular optical laryngoscopes can support effective skill acquisition and may reduce procedural variability during high-stress scenarios such as CPR [2,3]. Importantly, these potential benefits appear most pronounced under dynamic conditions rather than in controlled, static airway scenarios.

The optical design of these devices naturally promotes a midline approach, direct control of the epiglottis, and a more evident appreciation of airway anatomy skills that are central to pediatric intubation. Evidence from simulation studies, particularly those involving less experienced providers, suggests that training with tubular optical laryngoscopes can facilitate skill acquisition and help limit procedural variability during intubation under stressful conditions, such as CPR [41,42,43,44]. Seen in this light, tubular optical laryngoscopes can serve as a helpful adjunct in both training and clinical practice, particularly when preparing providers to manage pediatric airways during uninterrupted chest compressions. Their use in educational settings is best paired with structured, device-specific instruction and competency assessment, given the distinct way these devices are handled and the different visualizations they provide [45,46].

Introducing tubular optical laryngoscopes into pediatric resuscitation practice should follow the same evidence-based principles that guide the use of other airway technologies [47,48,49,50]. This includes prior validation in simulation settings, clearly defined training pathways, and regular performance evaluation once the device is in use. At present, however, most available data come from manikin and simulation studies. As a result, well-designed prospective clinical research will be needed to determine whether the procedural advantages observed in controlled settings translate into meaningful, patient-centered benefits during real pediatric CA.

Interpretation of these clinical and educational implications should also consider the level of operator experience and the potential influence of device-specific learning curves. Participants had no prior experience with the VieScope, and the results therefore reflect early-phase performance following structured introductory training rather than proficiency after extended clinical use. Although a standardized 60 min training session was provided to ensure baseline competence, learning effects cannot be fully excluded. It is plausible that performance with the VieScope would further improve with repeated use, as has been shown for other airway devices, while performance differences between devices may be less pronounced among experienced clinicians. Accordingly, the present findings should be interpreted as reflecting novice performance in a simulated pediatric resuscitation setting.

### 4.6. Strengths and Limitations

This study has several strengths, including a randomized crossover design that reduced inter-participant variability by allowing each participant to serve as their own control. At the same time, standardized scenarios with and without chest compressions enabled isolation of the “compressions effect”. In addition, outcomes combined objective endpoints (FPS, intubation time, Cormack–Lehane grade, and POGO score) with operator-perceived difficulty (ease of intubation and optimization maneuvers), providing a multidimensional assessment of device performance.

Several limitations also must be acknowledged. First, this was a manikin-based simulation study; real clinical airways involve secretions, bleeding, regurgitation risk, variable muscle tone, and anatomical heterogeneity that may materially affect comparative performance. Another significant limitation is that participants were PALS-trained medical students rather than seasoned pediatric airway clinicians. Consequently, the results primarily describe novice performance rather than outcomes achievable by more experienced providers. Third, only a single pediatric anatomical model (5-year-old) was studied, and generalization to infants or older children should therefore be made cautiously. Also, each laryngoscope scenario combination involved a single attempt per participant, limiting inferences regarding learning curves, skill retention, and performance after repeated practice. Additionally, continuous chest compressions were standardized using a mechanical compression device, which improved repeatability but may not fully reproduce the variability of manual compressions or the complexity of fundamental team dynamics during pediatric resuscitation. Although no intubation guides or stylets were used in this study, this approach was applied uniformly across all devices and therefore did not introduce systematic bias; nevertheless, future studies incorporating commonly used airway adjuncts may help further clarify their influence on device-specific performance.

### 4.7. Future Directions

There is also a clear rationale for expanding the anatomical scope of future research. Including a broader pediatric age range, from infants to older children, would enhance the generalizability of the findings [51,52,53,54]. Future work should take the present findings as a starting point and expand on them in a structured, but clinically grounded way. One obvious next step would be to involve experienced airway clinicians and full resuscitation teams, rather than individual novice operators [2,3]. This would make it possible to explore whether the performance differences observed here persist across different levels of expertise and in settings that better reflect real team-based resuscitation, including the interplay between the operator, the device, and overall cognitive and task load [2,3]. Another noticeable gap lies in age-related anatomy. Studying airway management in patients ranging from infants to older children would make future results easier to generalize [51,52,53]. In parallel, predefined difficult airway scenarios, such as limited mouth opening, an anteriorly positioned larynx, or airway contamination with blood or gastric contents, should be incorporated [55,56,57]. In particular, contaminated-airway models may help clarify whether the procedural advantages seen under controlled simulation conditions hold up in situations that more closely resemble real pediatric CA [58,59,60]. Additionally, comparative research should move beyond relying solely on conventional direct laryngoscopy. Rather than focusing on single devices in isolation, future studies could examine tubular optical laryngoscopes such as the VSL together with pediatric videolaryngoscopes and supraglottic airways. This side-by-side perspective would better reflect real clinical decision-making during pediatric CPR and help define the role of each device within airway algorithms [3]. Also, future investigations should incorporate resuscitation processes and system-level outcomes in addition to procedural endpoints [3,61]. Intubation success alone tells only part of the story. How airway management affects the overall course of resuscitation may be better captured by measures such as the time compressions are actually delivered, the duration of airway-related pauses, or the time to establish adequate ventilation [3,18]. Taking this broader perspective allows airway devices to be judged by their actual impact on the overall course of resuscitation, rather than by intubation success alone [62]. At the same time, simulation-based research remains an important starting point for hypothesis generation and controlled evaluation [63,64]. To move beyond this stage, prospective clinical studies will be needed. Observational registry analyses and pragmatic trials are likely to be key in determining whether the procedural gains demonstrated in simulation translate into tangible patient-centered outcomes, including oxygenation, return of spontaneous circulation, and neurological status at discharge [3,65]. In the absence of such data, any conclusions about clinical superiority should remain deliberately cautious [3].

## 5. Conclusions

In this simulated pediatric CA setting, the VSL demonstrated the most favorable overall performance compared with MAC and MIL direct laryngoscopes, with the differences most noticeable during uninterrupted chest compressions. These findings suggest that when pursuing tracheal intubation during pediatric resuscitation, selecting a device that enables rapid, stable visualization with minimal adjustment may enhance airway management and align with current guidelines, reducing interruptions in chest compressions. However, because this investigation was conducted using a pediatric manikin model, future clinical studies are necessary to establish the clinical relevance of these findings and their impact on patient-centered outcomes during pediatric cardiac arrest.

## Figures and Tables

**Table 1:** Intubation parameters in scenario without chest compressions.

Parameter	Intubation Technique		
	MAC	MIL	VSL
FPS, n (%)	37 (69.8%)	45 (84.9%)	53 (100%)
Time to intubation (s), mean (SD)	31.24 (9.95)	32.64 (9.31)	27.92 (9.18)
Cormack—Lehane grade, n (%)			
1	22 (41.5%)	26 (49.1%)	31 (58.5%)
2	27 (50.9%)	22 (41.5%)	21 (39.6%)
3	4 (7.6%)	5 (9.4%)	1 (1.9%)
4	0 (0.0%)	0 (0.0%)	0 (0.0%)
POGO Score, mean (SD)	83.36 (14.33)	87.38 (12.45)	86.17 (11.36)
Ease of intubation, mean (SD)	67.92 (15.96)	68.68 (19.04)	73.53 (15.67)
Optimization maneuvers, mean (SD)	1.15 (1.09)	0.92 (0.92)	0.68 (0.83)

Legend: FPS = First-Pass Success; MAC = the Macintosh laryngoscope; MIL = the Miller laryngoscope; POGO = Percentage of Glottic Opening; SD = Standard Deviation; VSL = the Bebé VieScope laryngoscope.

**Table 2:** Overall and post hoc comparisons of intubation performance parameters among the Macintosh (MAC), Miller (MIL), and VieScope (VSL) laryngoscopes in Scenario A (without chest compressions).

Parameter		Intubation Time	FPS	POGO Score	CL Grade	EOI	NOM
***p*** **-value**		0.040	0.032	0.346	0.161	0.189	0.202
Post hoc *p*-value	MAC vs. MIL	0.366	0.063	0.159	0.562	0.894	0.453
	MAC vs. VSL	0.111	0.017	0.367	0.056	0.083	0.153
	MIL vs. VSL	0.013	0.566	0.528	0.213	0.162	0.527

Legend: EOI = Ease of intubation; FPS = First-Pass Success; MAC = the Macintosh laryngoscope; MIL = the Miller laryngoscope; NOM = Number of Optimization Maneuvers; POGO = Percentage of Glottic Opening; VSL = the Bebé VieScope laryngoscope; CL = Cormack-Lehane grade.

**Table 3:** Intubation parameters in scenario with uninterrupted chest compressions.

Parameter	Intubation Technique		
	MAC	MIL	VSL
FPS, n (%)	28 (52.8%)	39 (73.6%)	45 (84.9%)
Time to intubation (s), mean (SD)	49.24 (16.05)	46.86 (14.08)	35.85 (12.95)
Cormack—Lehane grade, n (%)			
1	1 (1.9%)	3 (5.7%)	12 (22.6%)
2	12 (22.6%)	27 (50.9%)	30 (56.6%)
3	30 (56.6)	21 (39.6%)	10 (18.9%)
4	10 (18.9%)	2 (3.8%)	1 (1.9%)
POGO Score, mean (SD)	60.21 (20.39)	70.77 (19.89)	77.68 (17.56)
Ease of intubation, mean (SD)	42.72 (21.03)	53.72 (20.00)	64.32 (20.51)
Optimization maneuvers, mean (SD)	2.08 (1.12)	1.96 (1.26)	1.38 (1.15)

Legend: FPS = First-Pass Success; MAC = the Macintosh laryngoscope; MIL = the Miller laryngoscope; POGO = Percentage of Glottic Opening; SD = Standard Deviation; VSL = the Bebé VieScope laryngoscope.

**Table 4:** Overall and post hoc comparisons of intubation performance parameters among the Macintosh (MAC), Miller (MIL), and VieScope (VSL) laryngoscopes in Scenario B (with chest compressions).

Parameter		Intubation Time	FPS	POGO Score	CL Grade	EOI	NOM
***p*** **-value**		<0.001	<0.001	<0.001	<0.001	<0.001	0.035
Post hoc *p*-value	MAC vs. MIL	0.509	0.027	0.012	<0.001	0.006	0.392
	MAC vs. VSL	<0.001	<0.001	<0.001	<0.001	<0.001	0.019
	MIL vs. VSL	<0.001	0.151	<0.001	0.002	0.017	0.065

Legend: EOI = Ease of intubation; FPS = First-Pass Success; MAC = the Macintosh laryngoscope; MIL = the Miller laryngoscope; NOM = Number of Optimization Maneuvers; POGO = Percentage of Glottic Opening; VSL = the Bebé VieScope laryngoscope; CL = Cormack-Lehane grade.

## Data Availability

The data supporting the findings of this study are available from the corresponding author upon reasonable request (L.S.). The data will be available upon responsible request.

## Abbreviations

The following abbreviations are used in this manuscript:

AHA	American Heart Association
CA	Cardiac Arrest
CPR	Cardiopulmonary Resuscitation
FPS	First-Pass Success
MAC	Macintosh Laryngoscope
MIL	Miller Laryngoscope
PALS	Pediatric Advanced Life Support
POGO	Percentage of Glottic Opening
QCPR	Quality Cardiopulmonary Resuscitation
SD	Standard Deviation
VSL	Bebe VieScope Laryngoscope
